# Integrating UAV-Derived Diameter Estimations and Machine Learning for Precision Cabbage Yield Mapping

**DOI:** 10.3390/s25185652

**Published:** 2025-09-10

**Authors:** Sara Tokhi Arab, Akane Takezaki, Masayuki Kogoshi, Yuka Nakano, Sunao Kikuchi, Kei Tanaka, Kazunobu Hayashi

**Affiliations:** 1 Research Center for Agricultural Robotics, National Agriculture and Food Research Organization (NARO), Tsukuba 305-0856, Japan; 2 Institute of Agriculture Machinery, National Agriculture and Food Research Organization (NARO), Tsukuba 305-0856, Japan; 3 Central Region Agricultural Research Center, National Agriculture and Food Research Organization (NARO), Tsukuba 305-8666, Japan

**Keywords:** unmanned aerial vehicle, RGB, multispectral images, cabbage diameter, pose estimation, head fresh weight prediction, ML algorithms

## Abstract

Non-destructive diameter estimation of cabbage heads and yield prediction employing Unmanned Aerial Vehicle (UAV) imagery are superior to conventional approaches, which are labor intensive and time consuming. This approach assesses spatial variability across the field, effective allocation of resources, and supports variable application rates of fertilizer and supply chain management. Here, individual cabbage head diameters were estimated using deep learning-based pose estimation models (YOLOv8s-pose and YOLOv11s-pose) using high spatial resolution RGB images acquired from UAV 6 m during the cabbage-growing season in 2024. With a mean relative error (MRE) of 4.6% and a high mean average precision (mAP) 98.5% at 0.5, YOLOv11s-pose emerged as the best-performing model, verifying its accuracy for pragmatic agricultural use. The approximated diameter was then combined with climatic variables (temperature and rainfall) and canopy reflectance indices (normalized difference vegetation index (NDVI), normalized difference red edge index (NDRE), and green chlorophyll index (CIg)) that were extracted from the multispectral images with 6 m resolution and fed into AI models to develop individual cabbage head fresh weight. Among the machine learning models (MLMs) tested, CatBoost achieved the lowest Mean Squared Error (MSE = 0.025 kg/cabbage), highest R^2^ (0.89), and outperformed other models based on the Diebold–Mariano statistical test (*p* < 0.05). This finding suggests that an integrated AI-powered framework enhances non-invasive and precise yield estimation in cabbage farming.

## 1. Introduction

Smart agriculture is rapidly transforming traditional farming techniques by optimizing efficiency, maximizing production with minimum inputs, guaranteeing sustainability, and ensuring food security. Societies confronting aging agricultural communities urgently require effective and sustainable food supply management [1,2,3,4]. In this scenario, intelligent cultivation that integrates artificial intelligence, remote sensing, and data analytics has emerged as a promising solution to increase productivity while minimizing resource consumption.

Among these aforementioned advanced technologies, the application of deep learning techniques (DLTs) combined with UAV imagery for field-based, high-throughput, non-destructive phenotyping and crop yield prediction has recently increased [5,6,7]. These advanced technologies and tools enable the precise identification and monitoring of plant characteristics, such as size, shape, diameter, volume, and health state, through detection, segmentation, and classification, ultimately enabling accurate yield prediction and more efficient supply chain planning [8,9,10].

Recent studies have demonstrated the effectiveness of DLT in cabbage cultivation using UAV imagery by employing models such as DeepLab V3+, YOLOv5, Mask R-CNN, YOLOv8n-seg, UNet, and instance segmentation frameworks [9,11,12,13,14]. For instance, Kim et al. [11] developed a deep learning-based semantic segmentation framework to automate cabbage field identification in the highlands of South Korea. Similarly, a prior study proposed object segmentation on multispectral images and employed YOLOv5 to detect individual Chinese cabbage plants in UAV-derived RGB orthomosaic images [12]. Moreover, object-based image analysis (OBIA) and deep learning models such as Mask R-CNN have been applied to extract and count individual cabbage plants, with Mask R-CNN outperforming OBIA in terms of accuracy and robustness [9]. Additionally, CabbageNet, a model developed for real-time segmentation of cabbage heads, demonstrated the potential of intelligent automation in precision horticulture [13].

Despite these improvements in cabbage detection and segmentation, there is still a significant gap in the literature regarding precise cabbage diameter measurements for yield prediction using UAV-derived RGB and multispectral imagery. Majority of yield prediction methods rely on either physical sampling or indirect indicators, which restrict scalability and accuracy in commercial farming. These methods require considerable amounts of time and effort. However, drone multispectral and RGB sensors provide farmers with high-resolution images at a lower cost, making them more beneficial and readily available tools for precision farming [15,16,17].

The integration of UAV-derived RGB images with multispectral canopy reflectance indices and climatic variables (temperature and precipitation) into an AI model has remarkably improved crop feature extraction, biomass estimation, plant disease detection, and yield forecasting [18,19]. Generally used canopy reflectance indicators, such as the Normalized Difference Vegetation Index (NDVI), help estimate agricultural production [20]. Other indices such as NDRE and CIg also provide significant information on plant health, nutrition, canopy structure, stress, and chlorophyll content. This allowed the accurate phenotyping and monitoring of crop conditions in the field [21,22,23,24].

In addition to canopy reflectance indices, incorporating meteorological data derived from satellite remote sensing including LandSat8 land surface temperature (LST) and precipitation data from the Japan Aerospace Exploration Agency (JAXA), further strengthens the analytical foundation for precision agriculture. These climatic variables have been used in various studies on grapes, rice, maize, and cassava [25,26,27,28]. Furthermore, integrating vegetation indices with other crop parameters and plant characteristics obtained from RGB images help us to better understand agricultural situations at the field level. Additionally, combining this information with statistical and ML models enable the prediction of plant features and yields across many other crops, including fruits and vegetables [29,30].

Although UAV images and DLT are increasingly being utilized for crop monitoring and yield prediction, to date, no study has focused on estimating individual cabbage head weight directly from the diameter acquired from UAV-based RGB images. Cabbage is an economically valuable vegetable of which market price is largely determined by head size and homogeneity, qualities that are difficult to evaluate non-destructively at large scales. In this study, we have proposed a scalable, non-invasive method for estimating the fresh weight of cabbage heads in the field using vegetation indicators, climate data, and diameters obtained from high-resolution RGB UAV images. In contrast to conventional segmentation techniques, such as thresholding, edge detection, and region-based methods, which are commonly used to identify crop boundaries, this study employed a pose estimation (keypoint detection) model to estimate cabbage diameter [31]. In this context, pose estimation is a more efficient and accurate way to find certain geometric features, such as the outside edges of cabbage heads, which allow for accurate diameter measurement without requiring complete object segmentation. This innovative usage of poses enables a feature extraction approach that is both resilient and efficient, making it ideal for wide-field applications.

Therefore, the main goal of this study was to enable smart agricultural initiatives by building a deep learning-based system that employs a pose estimation model to recognize and locate individual cabbage heads in UAV RGB images. Specifically, the objectives of this study were to (1) estimate the cabbage head diameter from UAV RGB imagery using a pose estimation model; (2) predict individual cabbage head fresh weight using various ML algorithms by integrating head diameter, environmental data, and biophysical properties of cabbage; and (3) develop a spatial cabbage head fresh weight map based on the best-performing model output. This approach allows the precise, non-destructive, and large-scale measurement of cabbage yield and diameter estimation. Spatial yield maps provide vital information for harvest planning, resource allocation, and nutrient management. This improves both smart agriculture and data-informed decision-making in cabbage cultivation.

## 2. Materials and Methods

### 2.1. Experimental Field Summary and Cultivation Method

**Figure 1:**
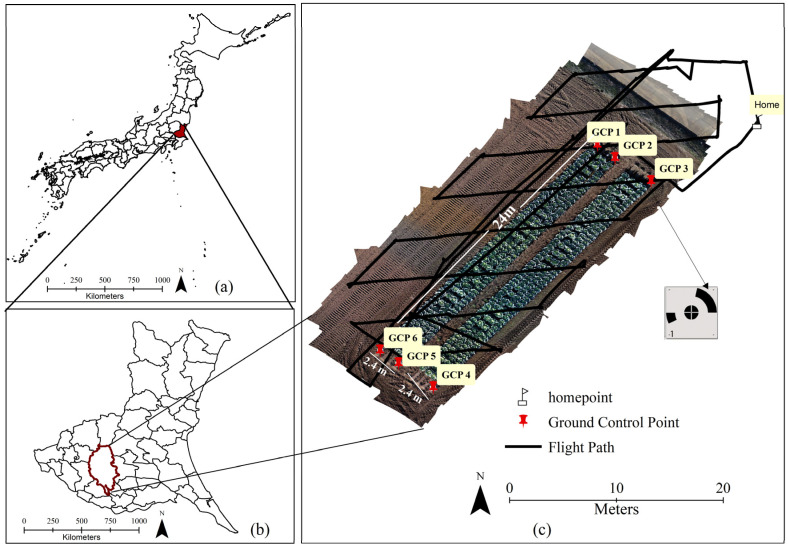
Geographical scope of the study: (**a**) location of Ibaraki Prefecture within the administrative boundaries of Japan, highlighting its regional context; (**b**) position of Tsukuba City within Ibaraki Prefecture; (**c**) location of the NARO experimental field, including the flight path, ground control points (GCPs), and home point.

The experiment was performed in a field of the National Agriculture and Food Research Organization (NARO) located in Tsukuba, Ibaraki Prefecture, Japan. The plots, covering an area of approximately 115.2 m^2^ (24 m × 4.8 m), are located at 36°1′30.53″ N latitude and 140°6′16.37″ E longitude (Figure 1). Three Japanese cabbage varieties (Renbu (TOHOKU SEED Co., Ltd., Utsunomiya, Japan), Tenku (TAKII SEED Co., Ltd., Kyoto, Japan), and Mikuni (KANEKO SEEDS Co., Ltd., Maebashi, Japan)) were transplanted on 4 September and 9 September 2024, with a total growth period of 128 days from seeding to harvesting (plot-1 Seeding on 7 August and harvesting on 13 December). A total of 60 cabbage seedlings were planted in each row. Each variety comprised 20 plants per row, yielding 160 cabbages per variety and 480 cabbages in total. The distance between plants was 0.4 m, and the distance between rows was 0.6 m. The harvest survey was conducted on 13 December and 16 December. During the surveys a total of 186 cabbages were randomly selected, labeled, placed in their designated bags, and transported to the laboratory under controlled conditions. Cabbage head diameter, total fresh aboveground weight, outer leaf weight, and head fresh weight were determined using quantitative assessments with a precision scale (A&D model) and a vernier caliper. The sample size was determined using a 95% confidence interval to ensure statistical reliability and representativeness of the collected data.

### 2.2. Data Acquisition

#### 2.2.1. Synopsis of Drone Images and Data Collection

During the cabbage-growing season, aerial photography was conducted using a DJI Phantom 4 Multispectral (P4M) drone on biweekly flights to evaluate and track the state of cabbage development with a total of 12 flights conducted. To measure cabbage head diameter and weight, high-resolution (6 m) RGB, and multispectral images were collected by using six ground control points (GCPs). These control points significantly improve data quality and contribute to more cost-effective surveys. Additionally, a Calibrated Reflectance Panel (CRP) was used following the DJI P4M guidelines to account for abnormalities caused by variations in lighting conditions during multispectral image capture. Images were captured under ideal lighting conditions, typically between 10:00 and 14:00 to ensure consistency and high-quality data. Moreover, each flight took about twenty minutes, and the drone was operated at six meters above ground with a ground pixel, resolution of 3.06 mm/pixel. This altitude allowed precise and detailed photography of the cabbage field. Agisoft Metashape software version 2.0.3 (https://www.agisoft.com) was used to mosaic approximately 300 original RGB images and 1500 multispectral images to produce an orthomosaic map of the entire field of the experiment. These efforts provide a fundamental basis for further research and applications in precision agriculture.

#### 2.2.2. Data Processing and Preparation for Cabbage Head Diameter Estimation Using Pose Estimation Techniques

**Figure 2:**
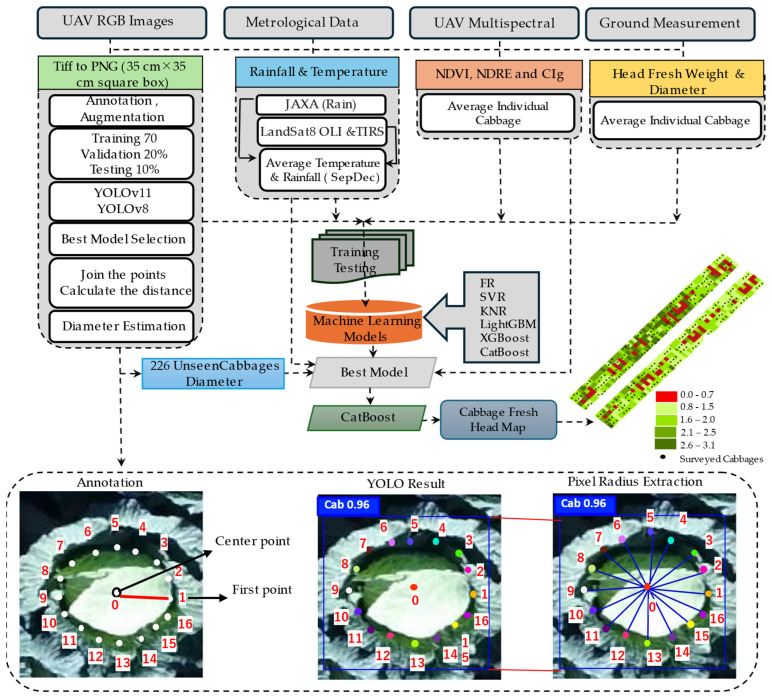
Schematic framework for estimating cabbage diameter and fresh head weight using RGB and multispectral UAV imagery combined with deep learning and machine learning techniques.

Individual photographs of cabbages extracted from the orthophoto-GeoTIFF images were selected for this study. Two orthophotos were taken during the harvest period, with dimensions of 6513 × 6072 pixels for plot-1 and 6380 × 5948 pixels for plot-2. Initially, the precise locations of the centers of each cabbage were identified using ArcGIS, with a spacing of 0.4 m between plants and 0.6 m between rows. A square-shaped polygon 35 cm × 35 cm graphic buffer was created around each cabbage. Square-shaped polygons were then applied to mask each cabbage, and the data format was changed from GeoTIFF to PNG. The original images, which had dimensions of 176 × 176 pixels, were first padded to 192 × 192 pixels and finally resized to 640 × 640 pixels before being fed into the YOLO-pose models. The dataset was divided into 70, 20, and 10% for training, validation, and testing, respectively. A Roboflow interface (https://roboflow.com/) was used to annotate the data. Different augmentation methods were used to expand the number of datasets, such as changing the hue and saturation (25%), adding noise (8.9%), changing the brightness (46%), adding blurring (10%), changing the exposure (15%), and converting the data to grayscale (25%). These augmentations resulted in a training dataset of 1217 images. Following this, the YOLO (You Only Look Once)-pose estimation model was employed to estimate the cabbage head diameter. To fully cover all cabbage heads, seventeen random keypoints (reference points), including one at the center of each cabbage, were manually labeled using RoboFlow. After augmentation, the dataset was expanded, and the cabbage-pose model (cab-pose) was trained on the training dataset and validated using the test dataset. Subsequently, the center point was joined to the remaining 16 keypoints to calculate the radius. The ground sampling distance (GSD), defined as the real-world distance represented by a single pixel, was approximately 0.306 cm/pixel in this study. The pixel distances from the center to each keypoint were averaged and then multiplied by the GSD. Finally, the cabbage head diameter was determined by doubling the average radius (Figure 2).

#### 2.2.3. Climatic Variables

Land surface temperature and precipitation were used to assess their effects on cabbage production. The Landsat 8 Operational Land Imager and Thermal Infrared Sensor (OLI and TIRS) bands were used for temperature estimation using the Google Earth Engine platform (https://earthengine.google.com/). A cloud mask was applied to the Landsat 8 Surface Reflectance (L8SR) data using the pixel quality assessment (QA) band [26,32,33]. During this procedure, bits three and five of the QA band, representing shadows and clouds, respectively, were examined. A clear-sky mask was established by setting both cloud shadows and flags to zero, signifying cloud-free scenes [33]. Furthermore, downscaling methods were used to downscale the spatial resolution of the Landsat 8 data to 10 m. Subsequently, we employed drone-derived NDVI, which was resampled in ArcGIS to match the desired spatial resolution [34]. The data from September to December 2024 were averaged, resulting in a final temperature map for the research area, created using ArcGIS Desktop 10.8.2. Temperature data was gathered for each cabbage location in the field with ArcGIS Desktop 10.8.2 (Esri, Redlands, CA, USA). Rainfall data were downloaded from the JAXA Global Rainfall Watch (GSMaP), including 12 h cumulative rainfall records collected from September to December 2024. Ten random points (x, y) were collected from the GSM and applied Inverse Distance Weighting (IDW) interpolation to generate rainfall maps for the entire field. These maps were then averaged and masked to each cabbage plot, providing a single rainfall value per cabbage. Seasonal averages of precipitation and temperature were utilized for the yield estimation in the machine learning models (https://global.jaxa.jp/, accessed on 2 September 2025) (Figure 2).

#### 2.2.4. Canopy Reflectance Indices

Multispectral imagery captured by the DJI P4 drone was used to compute critical canopy reflectance indices, including NDVI, NDRE, and CIg, which are essential for assessing plant growth and overall vitality. These indices provide valuable insights into the physiological status of crops and directly influence the accuracy of yield predictions. In this study, the P4 DJI drone equipped with a multispectral sensor captured data across specific spectral wavelengths: near-infrared (NIR) at 840 nm ± 26 nm, Red (RED) at 650 nm ± 26 nm, Green (G) at 560 nm ± 26 nm, and red edge (RE) at 730 nm ± 26 nm. Each of these bands serves a distinct purpose for evaluating different aspects of plant health (DJI, 2020) (Table 1) [35].

Consequently, the individual cabbage areas in the imagery were masked using certain indices (NDVI, NDRE, and CIg) in order to focus on specific plant regions [12,36,37]. The average values of each index for individual cabbage heads were then extracted and used as independent variables in the machine learning models aimed at predicting cabbage yield. By incorporating these vegetation indices, this study sought to enhance the precision and reliability of yield estimation by leveraging detailed spectral information provided by multispectral drone imagery (Figure 2).

### 2.3. AI Models for Cabbage Head Diameter Estimation

The YOLOv8s- and YOLOv11s-pose estimation models were used to determine the diameter of the cabbage head. Two dates—3 December and 9 December 2024—were selected for both fields (Plots #1 and #2), corresponding to the period when cabbage heads had reached their maximum growth stage before harvesting. The pose models were learned from the COCO dataset and the classification models were learned from the ImageNet dataset (https://github.com/ultralytics/ultralytics/issues/1915, accessed on 2 September 2025); https://docs.ultralytics.com/tasks/pose/, accessed on 2 September 2025). Pose estimation is the process of finding and locating keypoints in images to determine the arrangement of an object in space. Keypoints represent different parts of an object, such as joints, landmarks, and other important features. A set of two- or three-dimensional coordinates usually indicates the locations of these keypoints. Pose estimation models identify the essential points of an object and provide associated confidence scores. This method facilitates the identification of components within a scene and their spatial relationships (https://docs.ultralytics.com/tasks/pose/, accessed on 2 September 2025) [38,39].

### 2.4. Machine Learning Models for Cabbage Head Fresh Weight Prediction

In this experiment, ML models were used to estimate the fresh weight of the cabbage heads. Several vegetation reflectance variables (green vegetation index (GNDVI), soil-adjusted vegetation index (SAVI), modified soil-adjusted vegetation index (MSAVI), modified chlorophyll absorption reflectance index (MCARI), chlorophyll index based on the red band (CIred) and modified simple ratio index (MSRI)) showed substantial multicollinearity; in particular, those with correlation values over 90% were not included in the analysis and representative indices—NDVI, NDRE, and CIg were selected to reduce redundancy. For the head fresh weight prediction, the last set of predictor variables was the mean average per cabbage NDVI, NDRE, CIg, mean seasonal rainfall (average September–December), mean seasonal temperature (average September–December), and head diameter. Several ML models such as Random Forest (RF), Support Vector Regressor (SVR), K-Neighbors Regressor (KNR), Extreme Gradient Boosting (XGBoost), Light Gradient Boosting Machine (LightGBM), and Categorical Boosting (CatBoost) were among the many machine learning techniques used in this study. Fivefold cross-validation was performed to guarantee consistent performance ratings and prevent overfitting. A random search method was used to determine the best hyperparameters for the models [39] (Table 2). Finally, using a pose estimation model, the head diameters of 226 cabbage plants that were not explicitly recorded during the field survey were approximated to facilitate a thorough yield projection. The fresh weight of the head was computed using the expected diameter, along with other independent variables. The performances of the models were evaluated using the Diebold–Mariano test. Finally, ArcGIS Desktop 10.8.2 was used to create the final spatial map of cabbage head fresh weight. A 0.4 m × 0.6 m grid mesh was generated across the entire field using the Spatial Analyst tool. Subsequently, the predicted cabbage head fresh weight values were assigned to each grid cell, and the vector map was converted to a raster format representing cabbage head fresh weight in kilogram. The overall research schematic frameworks are explained in Figure 2.

### 2.5. Accuracy Assessment

#### 2.5.1. Head Keypoint Accuracy Assessment

The accuracy of the cabbage head diameter in the DLM was assessed using precision (P), recall (R), and mean average precision (mAP, calculated at the intersection across union criteria ranging from 50% to 95%) as assessment metrics to gauge the detection performance of the model for both boxes and poses [40,41]. The assessment metrics are stated as follows:

(1) $$P\;=\;\frac{TP}{\;TP\;+\;FP\;}$$

(2) $$R\;=\;\frac{TP}{\;TP\;+\;FN\;}$$

(3) $$\mathrm{mAP}=\frac{\sum_{i=1}^{N}\int_{0}^{1}P\left(R\right)\mathrm{dr}}{N}\times 100\%$$

where TP, FP, and FN denote the number of true positives, false positives, and false negatives, respectively. P and R refer to precision and recall. $\sum_{i=1}^{N}$ represents the average precision, while $\int_{0}^{1}P\left(R\right)dr$ calculates the area under the precision–recall curve from recall 0 to 1. N refers to the total number of classes, which in this study is 1, corresponding to the target class “cabbage head.”

#### 2.5.2. Cabbage Head Diameter and Fresh Weight Accuracy Assessment

The cabbage head diameter was evaluated using the mean absolute error and relative error. The assessment metrics were as follows:

(4) $$MAE=\frac{1}{n}\sum_{i=1}^{n}\left|y_{i}-{ỳ}_{i}\right|$$

where MAE is the mean absolute error (cm), n is the total number of cabbage head diameter observations, y_i_ is the actual (measured) diameter of the ith cabbage head, and ỳ_i_ denotes the predicted diameter of the ith cabbage head.

(5) $$RE=\frac{1}{n}\sum_{i=1}^{n}\left|\frac{{\;y}_{i}-{ỳ}_{i}}{y_{i}}\right|\times 100$$

where RE denotes the relative error (cm), n represents the total number of observations of cabbage head diameter, y_i_ is the actual (measured) diameter of the ith cabbage head, and ỳ_i_ is the predicted diameter of the ith cabbage head.

The machine learning models were evaluated based on R^2^ (the coefficient of determination) and root mean square error (RMSE). The equations are as follows:

(6) $$R^{2}=1-\frac{\;\sum_{i=1}^{n}{\left(y_{i}-{ỳ}_{i}\right)}^{2}\;}{\sum_{i=1}^{n}{\left(y_{i}-\mu \right)}^{2}}$$

(7) $$RMSE=\sqrt[{}]{\frac{1}{n}\sum_{i=1}^{n}{\left(y_{i}-{ỳ}_{i}\right)}^{2}}$$

where the R^2^ indicates percentage variation in fresh cabbage head weight as explained by the model. It ranges from 0 to 1. A score of 0 denotes a poor fit and 1 indicates that the model perfectly fits the data. The μ represents the mean. The RMSE indicates root mean square error which measures the average magnitude of prediction errors. This represents the typical difference between the measured y_i_ and the predicted fresh cabbage ỳ_i_ head weights.

#### 2.5.3. Performance Testing Using Diebold–Mariano Test

The Diebold–Mariano (DM) Test is a statistical technique employed to estimate the comparative prediction accuracy of two rival models. It assesses whether the differences in forecasting performance between models are statistically significant, based on a quantitative analysis framework. The DM statistic is calculated using the mean loss differential of the forecast errors from the two models, standardized by their estimated variance. A test is deemed significant when the *p*-value is less than the predetermined significance threshold (e.g., 0.05). In such instances, the null hypothesis is rejected, signifying that the prediction accuracy of the two models differs. A non-significant result indicates the absence of statistical evidence for a difference in predicting performance between the models. The efficacies of CatBoost with RF, CatBoost with XGBoost, and RF with XGBoost were evaluated by DM test. This test assists in identifying a model that yields accurate predictions. The DM formula is expressed as follows:

(8) $$DM=\frac{d_{mean}}{\sqrt[{}]{\frac{Vàr\left(d\right)}{T}}}$$

where DM signifies the Diebold–Mariano statistic, $d_{mean}$ indicates the mean difference in the forecast errors (loss differential), T represents the total number of observations, and Vàr(d) describes the variance of the loss differential [42,43,44].

### 2.6. Details of the Experimental Environment

In this study, the DL and ML models were executed in a system that was set up with the NVIDIA^®^ GeForce RTX 4070 SUPER GPU and an Intel(R) Core (TM) i7-14700KF CPU with 64 GB of RAM. This setup offers enhanced computational performance, facilitating efficient training and evaluation of models. Python 3.10.9 and PyTorch 2.5.1 were used for the configuration inside the Conda environment. To guarantee compatibility and the best performance for GPU-accelerated deep learning activities, the configuration also included CUDA 11.8 and cuDNN 8.8.0. Additionally, scikit-learn version 1.6.1 was used for the ML processes. This setup facilitates efficient model evaluation and training by improving performance.

## 3. Results

### 3.1. Cabbage Head Diameter Estimation Using Pose Estimation Model

**Figure 3:**
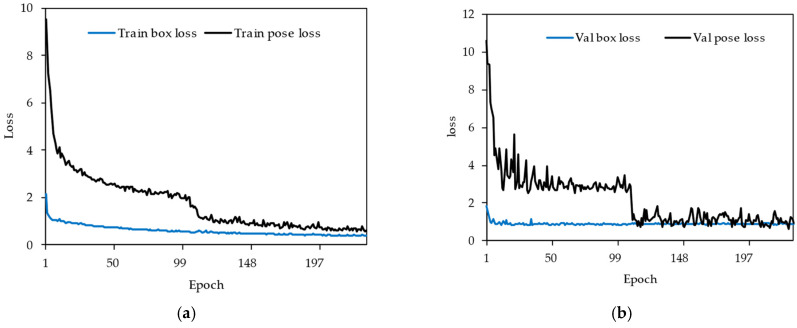

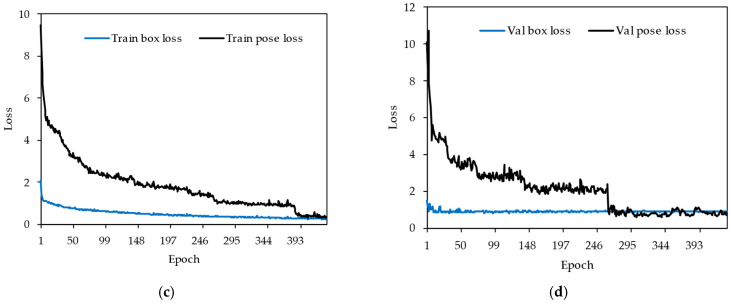
Loss trends across training epochs for both the YOLOv8s and YOLOv11s models: (**a**) YOLOv8s training losses for bounding box and pose estimation, (**b**) YOLOv8s validation losses for bounding box and pose estimation, (**c**) YOLOv11s training losses for bounding box and pose estimation, and (**d**) YOLOv11s validation losses for bounding box and pose estimation.

The models were trained for 800 epochs using the input images. The model had dimensions of 640 × 640 pixels and a batch size of 32 (Table 3). However, an early stopping strategy was applied, which automatically terminated training once the validation loss stopped improving. Additionally, the models were assessed over time in order to establish that both techniques progressively enhanced their capacity to identify keypoints around the cabbage head. The results indicated that in the YOLOv8s-pose model, the loss converged after approximately 120 epochs, and the training loss for the YOLOv11s-pose model stabilized after almost 400 epochs. For validation, the YOLOv11s model showed a steady loss after 250 epochs and the YOLOv8s model stabilized for approximately 120 epochs (Figure 3). This constant improvement in the detection accuracy during training revealed that the networks learned the spatial characteristics required for an exact CHD (cabbage head diameter) estimate.

**Figure 4:**
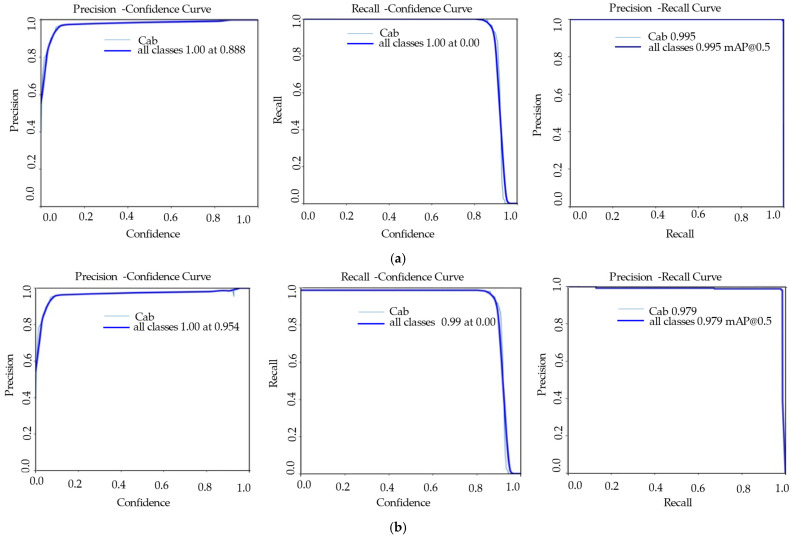
Performance evaluation parameters for the YOLOv8s-pose model: (**a**) precision, recall, and mAP for boxes; (**b**) precision, recall, and mAP for poses.

**Figure 5:**
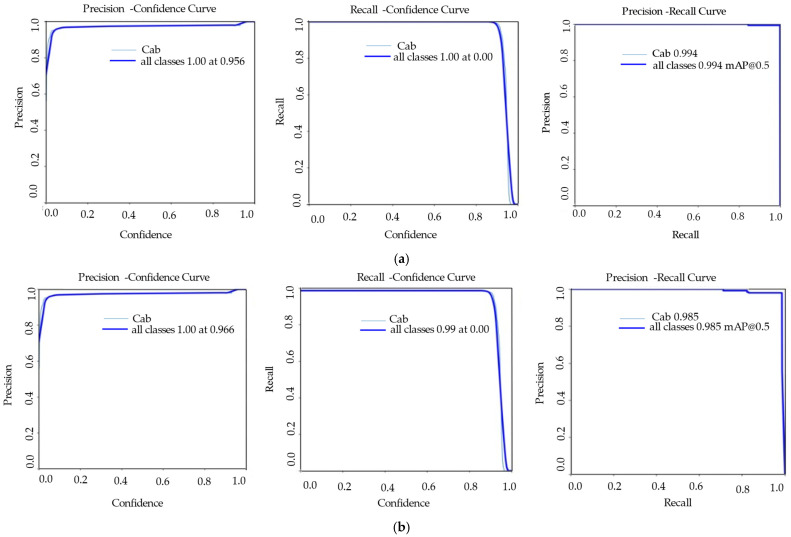
Performance evaluation parameters for the YOLOv11s-pose model: (**a**) precision, recall, and mAP for boxes; (**b**) precision, recall, and mAP for poses.

**Figure 6:**
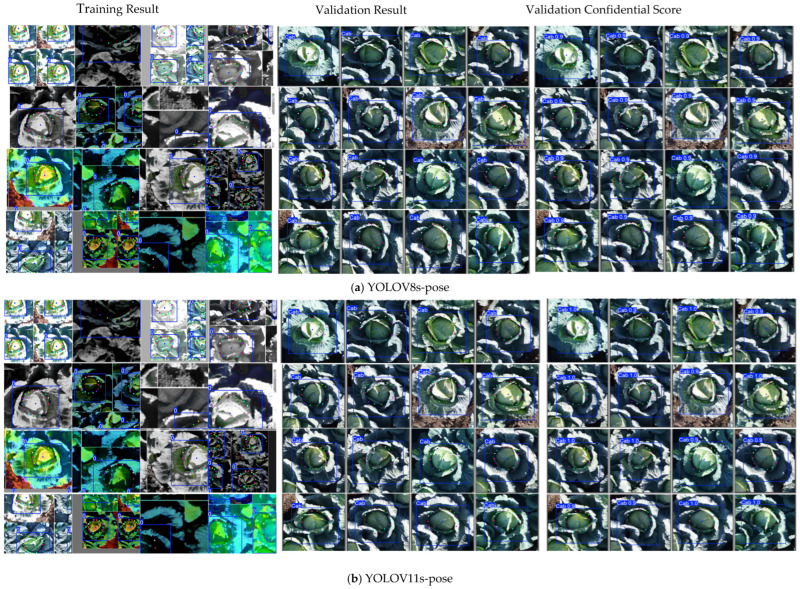

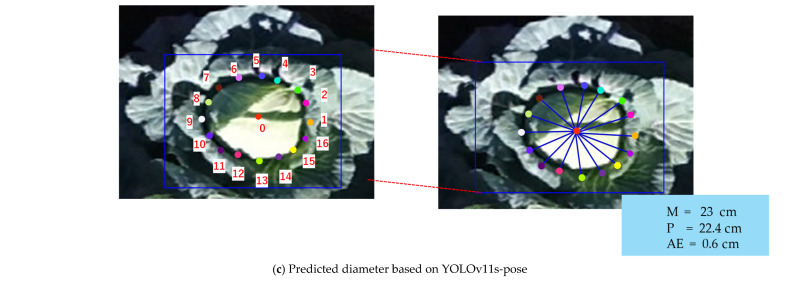
Training and validation results with confidence scores: (**a**) YOLOv8s-pose model performance during training and validation, including associated confidence scores; (**b**) YOLOv11s-pose model performance during training and validation, with corresponding confidence scores; and (**c**) cabbage head diameter (CHD) estimation result based on the YOLOv11s-pose model, illustrating the differences between predicted (P) and measured (M) values, along with the associated estimation error expressed as absolute error (AE).

YOLOv8s-pose model box detection attained a mean Average Precision (mAP@0.5) of 0.995, recall of 1.00, and precision of 0.89. Keypoint detection achieved a recall of 0.99, an mAP@0.5 of 0.98, and precision of 0.95 (Table 4, Figure 4). However, YOLOv11s box detection had a precision of 0.96, recall of 1.00, and mAP@0.5 of 0.99, whereas keypoint detection had an mAP@0.5 of approximately 0.99, a precision of 0.97, and a recall of 0.99 (Table 4, Figure 5). In conclusion, the results verified that the YOLOv11s-pose models generally showed improved stability and accuracy compared to YOLOv8s-pose models, which indicated a strong performance in estimating the cabbage head diameter (Figure 6a,b).

Following the identification of the best-performing model based on the validation criteria, particularly mAP@0.5, and loss plateauing behavior, the model was independently tested (Figure 6a,b). The test results showed that the highest confidence score was approximately 0.95, whereas the lowest score (recorded for only one image) was 0.80. This low confidence score caused the relative error to increase by approximately 12%, and the main reason was that part of the cabbage head was covered by leaves (Table 5). At this point, the expected performance could be assessed statistically. Specifically, 16 keypoints detected on each cabbage head were connected to the central reference point, and the average radius was calculated. This radius, initially measured in pixels, was then converted to real-world units (cm) and multiplied by two in order to obtain the final diameter for each cabbage (Figure 6c). The predicted cabbage head diameter showed a mean relative error (MRE) of approximately 4.6% across all the test samples, indicating high accuracy. This minimal error margin demonstrates the model’s efficiency and strong potential for real-world agricultural applications that require precise and automated measurements of the cabbage head diameter (Figure 6c, Table 5).

### 3.2. Cabbage Head Fresh Weight Prediction Using Machine Learning Models

**Figure 7:**
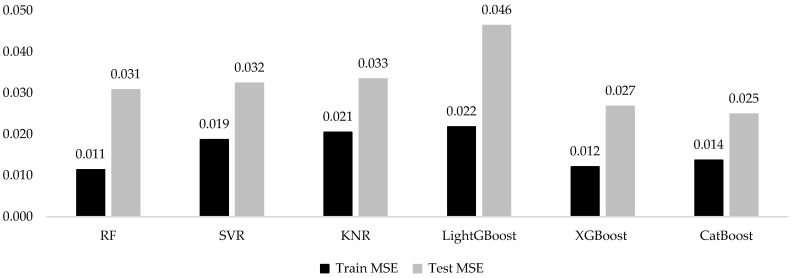
MSE of ML predicted models for training and testing phase.

The final set of explanatory variables included the mean individual cabbage NDVI, NDRE, CIg, cabbage head diameter, temperature, and rainfall; cabbage head fresh weight was designated as the response variable. Using field survey data, several machine learning (ML) models, including RF, SVR, KMN, LightGBM, XGBoost, and CatBoost, were developed, validated, and subsequently evaluated for previously unseen cabbage samples (Figure 7).

#### 3.2.1. Cabbage Head Fresh Weight Machine Learning Model Performance Evaluation Incorporating R^2^ and MSE

**Figure 8:**
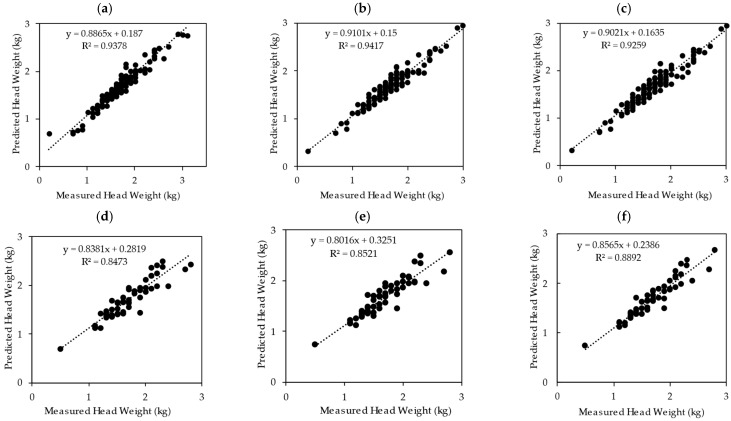
Comparison of regression performance among machine learning models in predicting cabbage head weight: (**a**) RF training, (**b**) XGBoost training, (**c**) CatBoost training, (**d**) RF testing, (**e**) XGBoost testing, (**f**) CatBoost testing.

The performance of several machine learning models regarding cabbage head fresh weight prediction is listed in Table 6. The RF and XGBoost R^2^ values were 0.94; for CatBoost, it was 0.93; for both SVR and KNR, they were 0.90; and LightGBM produced an R^2^ value of 0.88. However, the R^2^ value alone does not fully show how well the models perform, so other measures such as MSE were also considered during both the training and testing phases, as presented in Table 6 and Figure 7 and Figure 8. Furthermore, the MSE values for the training phase were RF (0.011 kg/cabbage), XGBoost (0.012 kg/cabbage), SVR (0.019 kg/cabbage), KNR (0.021 kg/cabbage), LightGBM (0.022 kg/cabbage) and CatBoost ( kg/cabbage 0.014). The corresponding MSE values for the testing phase were CatBoost (0.025 kg/cabbage), XGBoost (0.027 kg/cabbage), RF (0.031 kg/cabbage), SVR (0.032 kg/cabbage), KNR (0.033 kg/cabbage), and LightGBM (0.046 kg/cabbage). Among these models, CatBoost demonstrated the lowest MSE in the testing stage, including the best generalized performance in terms of prediction loss (Figure 7).

**Figure 9:**
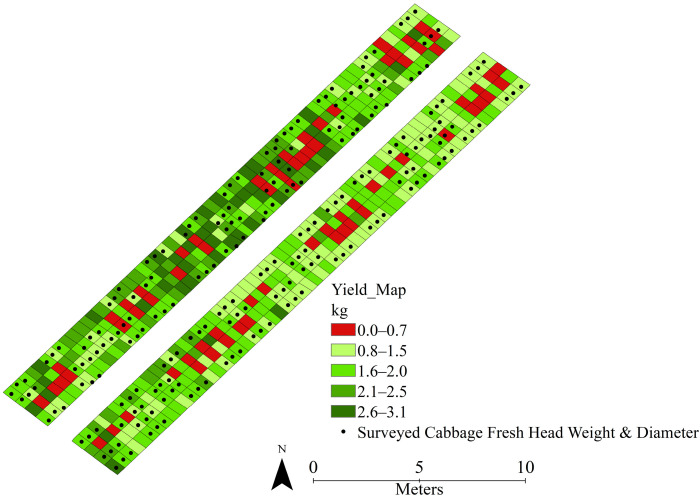
Spatial distribution map of cabbage head fresh weight (kg/cabbage) in the NARO experimental field.

Additionally, ArcGIS Desktop 10.8.2 (Esri, Redlands, CA, USA) was used to create a spatial prediction map of individual cabbage head fresh weights (kg per cabbage). The results indicated that the dark green areas in the produced spatially predicted map showed the highest yield—between 2.6 and 3.1 kg—whereas the red areas showed the lowest yield, between 0 and 0.7 kg (Figure 9).

#### 3.2.2. Evaluation of Best Model Performance (Random Forest, Extreme Gradient Boosting and Categorical Boosting) by Diebold–Mariano Statistical Test

This study assessed several MLMs based on their predicted efficacy. As shown in Table 7, CatBoost outperformed all the other models. The test results indicated that CatBoost had significantly fewer errors than both RF and XGBoost. Moreover, there was no significant difference in predictive performance between the RF and XGBoost models. Although RF and XGBoost exhibited the highest R^2^ values, their prediction errors were higher than those of CatBoost (Table 7).

## 4. Discussion

Deep learning models have been used to identify plant features, such as recognizing individual cabbages or drawing bounding boxes around them. They have also been used to predict the aboveground fresh weight of cabbage using RGB and multispectral images from cameras or UAVs [8,11,12]. Building on this foundation, our study advanced these approaches in two stages. In the first stage, a deep learning model was used to estimate the head diameters of individual cabbages. In the second stage, the diameter derived from the pose model was combined with multispectral and climatic data to accurately predict the fresh head weight of individual cabbages. These values reflect the physiological and environmental conditions of cabbage plants; thus, they help forecast the vital predictive ability of the head for fresh weight, which is crucial for supply chain management.

A comparison between deep learning models, namely the YOLOv11s-pose and YOLOv8s-pose, confirmed that the YOLOv11s-pose achieved better keypoint detection accuracy and greater model stability, with a low mean relative error (MRE) of 4.6% and a high mAP@0.5 of 98.5%. These results underscore the high potential of precision agriculture applications. These findings demonstrate that YOLOv11s-pose can successfully recognize and estimate spatial features from RGB images captured by UAVs, enabling agriculture to be monitored accurately and on a larger scale. These results matched recent improvements in orchard [41], in which YOLOv11-pose and vision transformer-based depth estimation models (such as Depth Anything V2) were very successful in estimating the 3D positions of young green apples. Whereas Depth Anything V2 produced the lowest RMSE (1.52) and MAE (1.28) for depth estimation [45], YOLOv11n obtained a pose accuracy of 0.92 and box accuracy of 0.91. These results demonstrate that YOLOv11 designs are useful for a wide range of horticultural tasks.

A recent study [46] presented a method for precise blueberry detection, 3D spatial localization, and pose estimation using visual perception with the YOLOv11 model. The experimental results demonstrated a high detection accuracy of 95.8% mAP50-95, a positioning error of 7.2 mm within 0.5 m, and an average pose error of 19.2° [46]. Although another study focused on the robotic thinning of green apples, it highlighted the strong cross-application potential of artificial intelligence by using similar AI models for yield prediction in open-field vegetable crops [41]. Overall, these studies have pointed to a growing trend toward non-invasive, AI-enabled crop-monitoring systems that can drastically reduce labor needs, greatly increase the spatial resolution of measurements, and improve decision-making in field-based production.

Among the machine learning models evaluated in our study, the CatBoost model emerged as the most effective, with the lowest test-phase mean squared error (MSE) of 0.025 kg/cabbage and a strong R^2^ value of 0.89. Random Forest and XGBoost achieved slightly lower R^2^ values test-phase (both testing 0.85) (Figure 8), and the Diebold–Mariano test confirmed that CatBoost’s predictions were statistically more accurate (*p* < 0.05), illustrating its strength in handling complex feature interactions in agricultural data (Table 7). This finding is consistent with previous studies [47,48,49], who combined satellite data, applied the Random Forest, Support Vector, and CatBoost models and obtained an R^2^ of 0.95 and an MAE of 0.31 for the CatBoost model [47]. Climate and pesticide data utilized by [48] were also employed with CatBoost for rice yield prediction, achieving an R^2^ of 0.80 and RMSE of 0.24 [48]. Another study reported that CatBoost outperformed both LightGBM and XGBoost in predicting eggplant yield [49]. These consistent results highlight CatBoost’s strength in modeling agricultural yields because it was developed based on a mechanism called ordering boosting, which makes it resistant to overfitting. This feature enables the model to make better predictions and generalizations.

The primary limitation of this study is that only three Japanese cabbage varieties, Ranbu, Tenku, and Mikuni, were used to estimate cabbage diameter. Our next step is to apply the model to additional Japanese cabbage varieties, which will allow us to validate its generalizability and robustness for future studies. Furthermore, in the field, we plan to implement variable-rate fertilizers using the yield map developed in this study. By comparing the next year’s yield map with the current yield map at the end of the maturity stage, we aim to assess temporal yield variability and evaluate the impact of variable-rate input techniques on cabbage production.

Overall, our research findings have conclusively shown that a framework using AI, and an integrated UAV is applicable for precision vegetable cultivation. This technique enhances decision-making in the supply chain by delivering precise and spatially explicit yield predictions. This study advances the evolution of agriculture using intelligent data-driven approaches that promote sustainable and efficient crop management.

## 5. Conclusions

This study has revealed how well computer vision and machine learning technique (MLT) can be combined to accurately estimate cabbage head diameter and predict fresh weight. Pose estimation models were tested in this research, and YOLOv11s-pose stood out for its stability and higher accuracy compared to YOLOv8s-pose, achieving strong keypoint detection performance and high mAP@0.5 scores. The diameter estimated using YOLOv11 had a low MARE of 4.6%, confirming the reliability of the model for practical use in agriculture. Moreover, the head fresh weight was predicted using machine learning models incorporating climatic and agronomic features. CatBoost emerged as a superior machine learning model, with the lowest mean squared error (MSE) of 0.014 for training and 0.025 for testing, and R^2^ values of 0.93 for training and 0.89 for testing, respectively. While Random Forest and XGBoost had higher R^2^ values during training (0.94), their performance during testing was worse, yielding R^2^ values of 0.85 and elevated MSEs of 0.011 kg (RF training), 0.031 kg (RF testing), 0.012 kg (XGBoost training), and 0.027 kg (XGBoost testing). ArcGIS software was used to map spatial variations in cabbage production and to further use the map for precise production. The findings indicate that integrating artificial intelligence methodologies with UAV footage offers a non-destructive, precise, and scalable approach to monitor cabbage diameters and predict yields in actual agricultural settings.

## Figures and Tables

**Table 1:** Specifications of the Unmanned Aerial Vehicle (UAV).

Item	Specification
UAV type	DJI Phantom 4M Drone
Camera sensor	RGB and multispectral images
Working frequency	2.400–2.483 GHz
Filters	Blue (B): 450 nm ± 16 nm; Green (G): 560 nm ± 16 nm, Red (R): 650 nm ± 16 nm; red edge (RE): 730 nm ± 16 nm, near-infrared (NIR): 840 nm ± 26 nm.
Lenses	FOV (Field of View): 62.7° Focal Length: 5.74 mm (35 mm format equivalent: 40 mm), autofocus set at ∞ Aperture: f/2.2
Battery	6000 mAh LiPo 2S
Maximum takeoff weight	1487 g
Maximum flight time	Approx. 27–28 min

**Table 2:** Selected parameters for the ML models.

Model	Hyperparameter	Value
Random Forest (RF)	n_estimators	184
	max_depth	21
	random_state	42
	min_samples_split	8
	min_samples_leaf	2
Support Vector Regressor (SVR)	Gamma	Scale
	kernel	rbf
	C	2
K-Neighbors Regressor (KNR)	n_neighbors	5
Light Gradient Boosting Machine (LightGBM)	n_estimators	150
	max_depth	6
	random_state	42
	Learning_rate	0.045
Extreme Gradient Boosting (XGB)	n_estimators	375
	max_depth	1
	random_state	42
	Learning_rate	0.07
Categorical Boosting (CatBoost)	n_estimators	400
	max_depth	1
	random_state	42
	Learning_rate	0.08

**Table 3:** Training parameters for the cabbage head diameter estimation.

Model	Input Image Dimensions	Batch Size	Epoch
YOLOv8	640 × 640	32	800
YOLOv11	640 × 640	32	800

**Table 4:** Pose estimation best result for the boxes and pose.

Model	Parameter	Precision	Recall	Mean Average Precision–Recall (mAP)@0.5
YOLOV8s	Boxes	0.888	1.00	0.995
	Pose	0.954	0.99	0.979
YOLOv11s	Boxes	0.956	1.00	0.994
	Pose	0.966	0.99	0.985

**Table 5:** Test results for predicted cabbage head diameter (cm), including mean absolute error (MAE) and mean absolute relative error (MARE).

No.	Variety	Measured CHD * (cm)	Average Radius (cm)	Predicted CHD * (cm)	Absolute Error (cm)	Relative Error (%)
1	Tenku	23.0	11.2	22.4	0.6	2.7
2	Renbu	21.2	10.89	21.78	0.58	2.6
3	Tenku	21.2	11.42	22.84	1.64	7.5
4	Mikuni	20.9	10.69	21.39	0.49	2.2
5	Renbu	20.9	10.69	21.38	0.48	2.2
6	Renbu	21.2	9.92	19.84	1.36	6.2
7	Renbu	20.8	11.28	22.56	1.76	8.0
8	Renbu	20.0	10.13	20.25	0.25	1.1
9	Renbu	21.6	11.64	23.27	1.67	7.6
10	Mikuni	24.05	10.69	21.38	2.67	12.1
11	Mikuni	22.45	11.14	22.28	0.17	0.8
12	Tenku	23.7	10.57	21.14	2.56	11.6
13	Renbu	22.95	11.33	22.67	0.28	1.3
14	Tenku	24.3	11.96	23.92	0.38	1.7
15	Tenku	22.1	12.36	24.73	2.63	12.0
16	Tenku	23.15	11.80	23.60	0.45	2.0
17	Renbu	21.55	10.89	21.77	0.22	1.0
18	Tenku	22.9	11.65	23.30	0.4	1.8
19	Renbu	21.75	11.47	22.95	1.2	5.5
20	Tenku	23.5	11.52	23.03	0.47	2.1
21	Mean				10.1	4.6

* CHD means cabbage head diameter. Average Radius × 2 = Predicted CHD.

**Table 6:** Coefficient of determination (R^2^) and mean squared error (MSE) of machine learning models during training and testing phases.

Model	Train		Test	
	R^2^	MSE	R^2^	MSE
RF	0.938	0.011	0.847	0.031
SVR	0.904	0.019	0.830	0.032
KNR	0.896	0.021	0.813	0.033
LightGBM	0.884	0.022	0.751	0.046
XGBoost	0.942	0.012	0.852	0.027
CatBoost	0.925	0.014	0.889	0.025

**Table 7:** Performance evaluation of CatBoost, XGBoost, and RF by Diebold–Mariano test for comparison.

Comparison	DM Type	DM Statistic	*p*-Value	Significance (*p* < 0.05)	Better Model
RF vs. XGBoost	Squared Errors	0.4756	0.6344	Not Significant	-
CatBoost vs. RF	Squared Errors	2.7514	0.0059	Significant	CatBoost
CatBoost vs. XGBoost	Squared Errors	2.66	0.0078	Significant	CatBoost
RF vs. XGBoost	Absolute Errors	1.2266	0.22	Not Significant	-
CatBoost vs. RF	Absolute Errors	2.9753	0.0029	Significant	CatBoost
CatBoost vs. XGB	Absolute Errors	2.0725	0.0382	Significant	CatBoost

## Data Availability

Access to the data used in this study is restricted due to licensing agreements. As such, the data is not publicly available. However, they may be obtained from the corresponding author upon reasonable request and with appropriate permission.

## Abbreviations

The following abbreviations are used in this manuscript:

UAV	Unmanned aerial vehicle
YOLO	You only look once
MARE	Mean absolute relative error
NDVI	Normalized difference vegetation index
NDRE	Normalized differences red edge index
CIg	Green chlorophyll Index
MLMs	Machine learning models
DLT	Deep learning techniques
LST	Land surface temperature
JAXA	Japan aerospace exploration agency
CRP	Calibrated reflectance panel
OLI	Operational land imager
TIRS	Thermal infrared sensor
QA	quality assessment
GNDVI	Green normalized vegetation index
SAVI	Soil-adjusted vegetation index
MCARI	Modified chlorophyll absorption reflectance index
MSAVI	Modified soil-adjusted vegetation index
RF	Random forest
SVR	Support vector regressor
KNR	K-neighbors regressor
XGBoost	Extreme gradient boosting
LightGBM	Light gradient boosting Machine
CatBoost	Categorical gradient boosting
DM	Diebold–Mariano
mAP	Mean average precision
CHD	Cabbage head diameter
